# The role of lipids in the inception, maintenance and complications of dengue virus infection

**DOI:** 10.1038/s41598-018-30385-x

**Published:** 2018-08-07

**Authors:** Carlos Fernando Odir Rodrigues Melo, Jeany Delafiori, Mohamad Ziad Dabaja, Diogo Noin de Oliveira, Tatiane Melina Guerreiro, Tatiana Elias Colombo, Maurício Lacerda Nogueira, Jose Luiz Proenca-Modena, Rodrigo Ramos Catharino

**Affiliations:** 1 INNOVARE Biomarkers Laboratory, School of Pharmaceutical Sciences, University of Campinas, Campinas, Brazil; 2 School of Medicine from São José do Rio Preto (FAMERP), São José do Rio Preto, Brazil; 3 Laboratory of Study of Emerging Viruses (LEVE), Department of Genetic, Evolution and Bioagents, Institute of Biology, University of Campinas, Campinas, Brazil

## Abstract

Dengue fever is a viral condition that has become a recurrent issue for public health in tropical countries, common endemic areas. Although viral structure and composition have been widely studied, the infection phenotype in terms of small molecules remains poorly established. This contribution providing a comprehensive overview of the metabolic implications of the virus-host interaction using a lipidomic-based approach through direct-infusion high-resolution mass spectrometry. Our results provide further evidence that lipids are part of both the immune response upon Dengue virus infection and viral infection maintenance mechanism in the organism. Furthermore, the species described herein provide evidence that such lipids may be part of the mechanism that leads to blood-related complications such as hemorrhagic fever, the severe form of the disease.

## Introduction

Dengue virus (DENV) is an arbovirus transmitted by mosquitoes of the genus *Aedes*, such as *Aedes aegypti* and *Aedes albopictus*. DENV is associated with outbursts of febrile diseases in the tropics since the 80’s^1^. The large number of DENV-infected patients every year, estimated by the World Health Organization in 390 million, makes DENV the most hazardous arbovirus in the world.

DENV is a series of enveloped viruses belonging to the family *Flaviviridae*, genus *Flavivirus*, which are classified in four closely related and antigenically distinct serotypes (DENV-1, DENV-2, DENV-3 and DENV-4). Similary to other flavivirus, the DENV genome consist of a single-stranded positive sense RNA (ssRNA) of almost 11 kb, which encodes a polyprotein that is cleaved into three structural proteins (the capsid (C), the pre-membrane (prM) and the envelope (E) and seven nonstructural proteins, named NS1, NS2A, NS2B, NS3, NS4A, NS4B, and NS5^2^.

Although the spectrum of clinical outcomes of patients’ responses to DENV varies from a subclinical infection to death, the majority of symptomatic patients develop an acute, self-limiting febrile manifestation. Lasting approximately 4–7 days, it is characterized by fever, chills, retro-orbital headache, myalgia, malaise, leukopenia, thrombocytopenia (sometimes severe) and elevated levels of hepatic transaminases^3^. In contrast, a small percentage of infected patients, usually children or adults during a second infection with a different DENV serotype, may develop severe dengue, characterized by spontaneous bleeding, plasma leakage, shock, and organ failure^3^.

The available knowledge indicates that the outcome of DENV infection depends on several factors produced during the beginning of the viral infection such as viral load, presence of non-neutralizing antibodies, immune cells recruitment and production of immune mediators^2^. These factors determine whether the environment is favorable or unfavorable for disease progression by either controlling the viral infection or impairing inflammatory reaction, associated with vascular permeability. Nevertheless, the lack of reliable immunological and other metabolic markers for either protective or pathological responses still an important gap that hinders the development of new diagnostic or prognostic tests or vaccine candidates^4,5^.

Within this context^4,5^, this work aimed to verify the changes in serum lipidome of patients infected with DENV-4, since lipids have been shown to be of great importance in the viral infection process^6–8^. Although the lipid profile of patients infected with DENV has already been established in other studies, most of them performed using liquid chromatography coupled to mass spectrometry (LC-MS) techniques^9–11^, whereas the present study used no chromatographic approach. We intended to analyze samples with the least possible preparation and manipulation, attempting to minimize as much as possible changes in the biological matrix used. Additionally, direct infusion high-resolution mass spectrometry allows us to analyze a wide range of lipids, a characteristic that is impaired when using LC-MS, since the column separates lipids by their respective physicochemical characteristics such as polarity, isomerism and others^12,13^.

## Metabolomic approach of serum of patients infected with DENV-4

**Figure 1:**
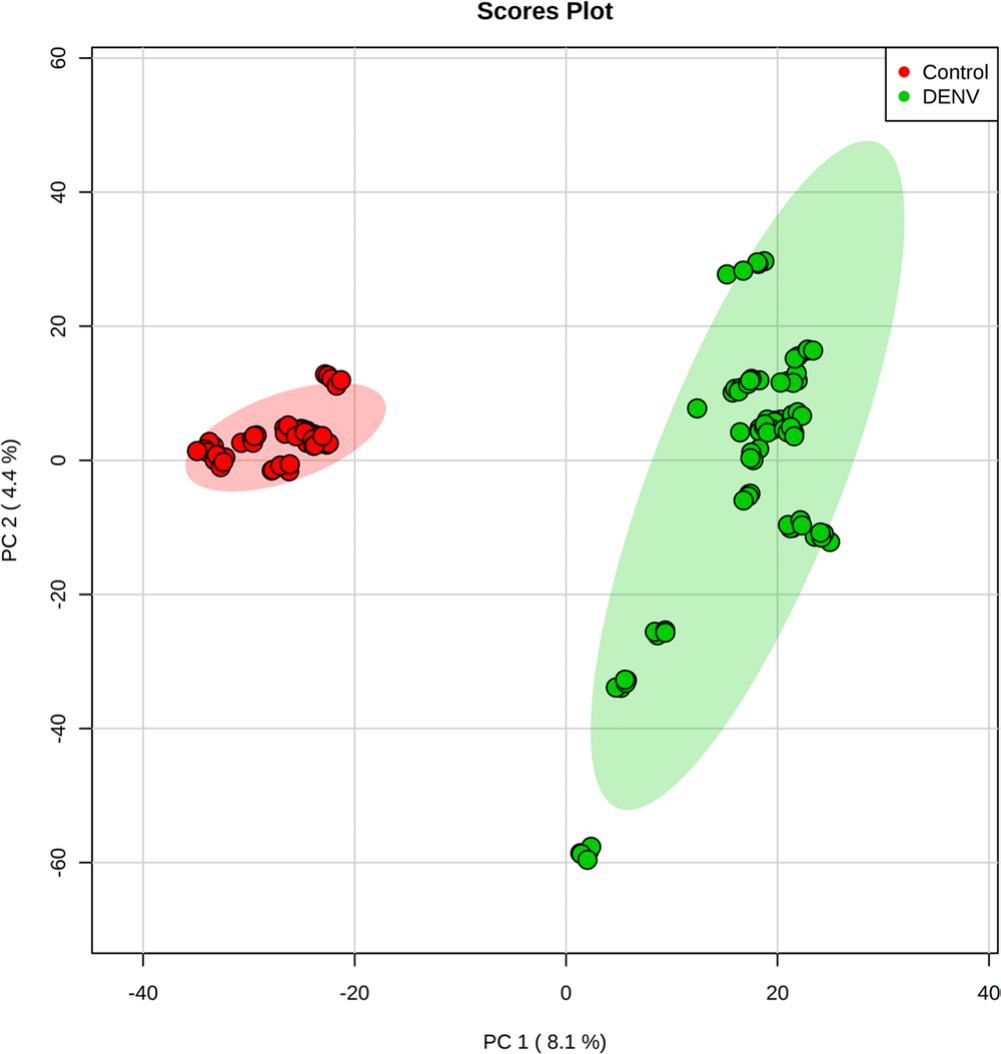
Scores plot between the first two principal components (PCs) selected from the Principal Component Analysis.

**Figure 2:**
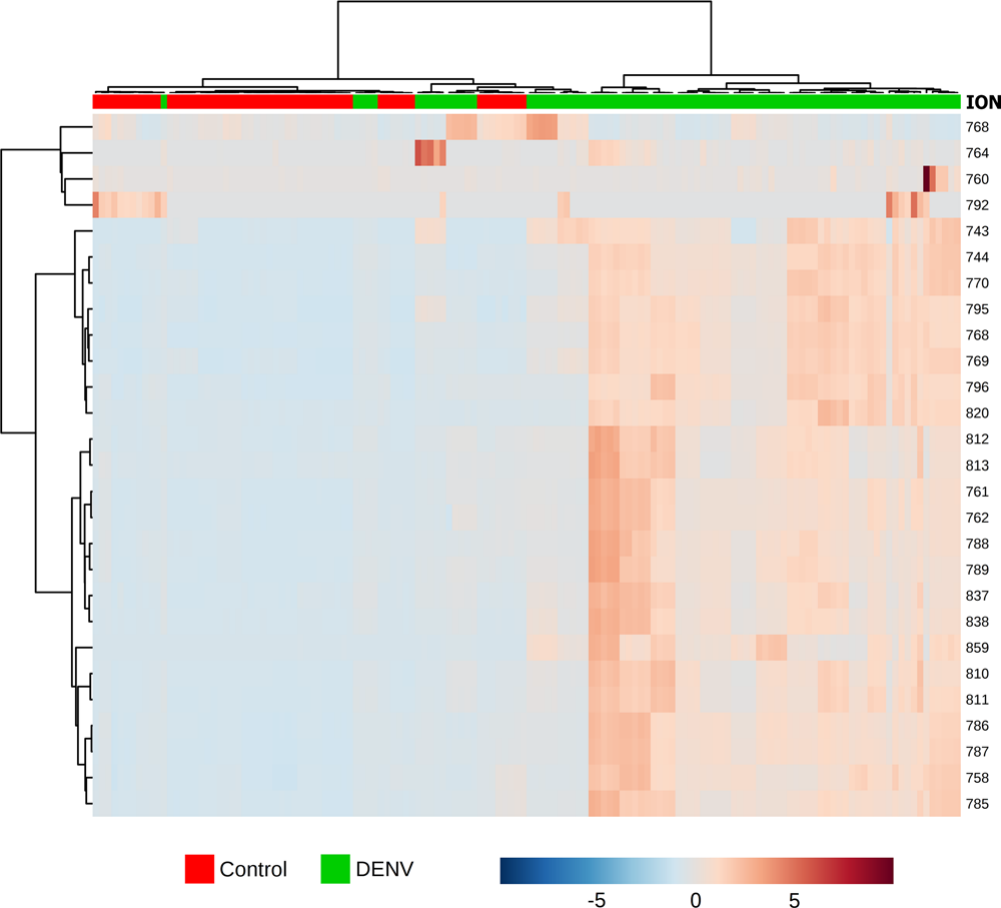
Clustering result for the 27 top features selected by Principal Component Analysis shown as a heatmap (distance measured by Pearson’s distance measurement and Ward’s clustering algorithm). The color-coded thermometer (bottom) indicates the relative presence of metabolites among the groups.

PCA clearly shows the separation between Control and DENV-infected patients, as shown in Fig. 1. This multivariate data analysis method was chosen because it is an unsupervised approach, capable of reducing the number of variables (reduction of dimensions) in the original dataset (raw data) based on the similar features between the samples, helping to find the most representative variables (features) responsible for each of the two clusters formed^14^, according to Fig. 1. This enabled the election of a feature set that is characteristic for each analyzed group, namely DENV-infected patients and healthy individuals. The bidimensional score plot in Fig. 1 is derived from the analysis performed with the data collected by mass spectrometry in the positive mode; from this clustering, we selected and characterized the features (potential metabolomic markers) that presented the greatest relevance in discriminating both groups. To illustrate the characteristic markers chosen by PCA, a heatmap of all features selected by this model was built using Pearson’s distance measurement and Ward’s clustering algorithm (Fig. 2).The Fig. 2 clearly illustrates the differences in the pool of metabolites between both analyzed groups. Following structure elucidations by mass accuracy and MS/MS reactions^15^ (Supplementary Information 1), three precursors of Platelet Activation Factor (PAF) [*m/z* 768.5917, *m/z* 770.6043 and *m/z* 792.5917], three Phosphatidylcholine derivatives (PC) [*m/z* 762.6022, *m/z* 838.6336 and *m/z* 796.6231] and four triglycerides [*m/z* 743.6169, *m/z* 769.6327, *m/z* 795.6412 and *m/z* 859.7765] were identified as characteristic for the DENV group; a list of characterized molecules is available in Table 1. Moreover, by elucidating relevant species such as the above mentioned, this work has confirmed that it is possible to identify *in vivo* which are the lipids associated with the phenotype of viral infection process by directly infusing the serum of infected patients in an HRMS instrument, regardless of any previous chromatographic approach.

**Table 1:** Lipid markers elected by Principal Component Analysis from the serum of patients infected with DENV (DENV group).

Exact mass	Theoretical Mass	Error (ppm)^a^	MS/MS fragmentation	Adducts	Platform^b^	Molecule	Log2 (FC)^c^
743.6169	743.6184	2.0172	684, 619, 555, 487	[M + H]+	MID 98508	TG(44:4)	4.245
					MID 99076		
769.6327	769.6341	1.8190	709, 645, 581, 587	[M + H]+	MID 99084	TG(46:5)	2.9707
					MID 98516		
795.6412	795.6424	1.5082	736, 612, 607, 590	[M + H]+	MID 99092	TG(48:6)	3.7961
					MID 98531		
859.7765	859.7749	1.8610	799, 676, 842, 671	[M + H]+	MID 4798	TG(52:2)	7.4812
762.6022	762.6007	1.9670	575,704, 621, 719	[M + H]+	MID 59328	PC(34:0)	2.6625
					MID 39142		
					MID 59482		
					MID 59708		
					MID 39823		
784.5836	784.5851	1.9118	579, 595, 725, 601	[M + H]+	MID 59843	PC(36:0)	8.9199
					MID 59614		
838.6336	838.6320	1.9079	779, 649, 655, 721	[M+H]+	MID 59917	PC(40:0)	4.8301
					MID 59982		
					MID 39855		
768.5917	768.5902	1.9516	709,581, 585, 563	[M+H]+	MID 43414	PC(O-36:4)	3.2534
					MID 40083		
					MID 76437		
770.6043	770.6058	1.9465	711,583, 726, 567	[M+H]+	MID 76435	PC(O-36:3)	3.6014
					MID 40080		
					MID 43415		
792.5917	792.5902	1.8925	733, 416, 609, 605	[M+H]+	MID 62936	PC(O-38:6)	2.7396
					MID 40092		
796.6231	796.6215	2.0085	737, 613, 778, 752	[M+H]+	MID 76462	PC(O-38:4)	2.9874
					MID 76423		
					MID 40129		

^a^Error = ((Exact Mass-Theoretical Mass)/Exact mass) * 10^6^.

^b^METLIN ID^15^.

^c^Log_2_ (FC): Log2 transformed fold change where FC = Fold Change (DENV/Control) prior normalization.

## Compounds involved in the pathophysiological mechanism of DENV-4 infection

Our findings corroborate previous reports by other groups, in studies that identified the production of a series of polypeptides that act as inflammatory mediators during the immune response of the host^3^, participating in the autophagy process by signaling between virus and host cell^2^ and in their replication process^16^. These studies have also reported the synthesis of lipid mediators during viral infection, which are related to the signaling, control, and maintenance of both the immune response and DENV pathogenesis^6,17–19^.

## Phosphatidylcholines (PC) and Triglycerides (TG)

The importance of lipid changes during DENV cell infection is evident when the virus assumes control of the cellular metabolism by controlling and regulating autophagy mechanisms to meet the needs of the viral replication^20^.

Autophagy is a general term used to refer to pathways by which cytoplasmic material (soluble macromolecules and organelles) are delivered to the lysosomes for degradation^21^. There are three different processes characterized by autophagy: macroautophagy, autophagy mediated by chaperones, and microautophagy^21^. By these processes, an eukaryotic cell is able to promote essential lysosomal degradation for survival, differentiation, development and homeostasis, presenting an important adaptive role in the protection of organisms against several pathologies^22^. Eliminating defective proteins and organelles with the potential to trigger pathogenic processes prevents the abnormal accumulation of protein aggregates and the removal of obligate intracellular parasites (OIPs); additionally, the autophagic process also plays an important role in the innate and adaptive immunity: it is responsible for the formation of epitopes presented by MHC complexes^23^. Autophagy is rapidly and positively regulated by cells that need to obtain intracellular nutrients, either during a period of nutrient deprivation or absence of growth factors, as well when there is high energy demand^22^. In this way, DENV, like any other virus, controls the cellular mechanisms in its favor^20^. By taking control of the autophagy processes, the virus is able to control cellular lipid metabolism^24^, providing the demands required in the viral infection process^20^.

Viral growth occurs through the formation of viral replication complexes (VRCs)^25^, consisting of lipid vesicles constructed by all positive-strand RNA viruses from the reorganization of the host intracellular membranes; within this vesicle, viral assembly occurs^26^.

Zhang, Jiantao, *et al*. (2016) demonstrated that a significant increase in PC is associated with viral replication, and occurs mainly in the perinuclear membrane of the endoplasmic reticulum (ER), where viral replication occurs; additionally, their data showed that PC accumulation is due to the formation of this lipid class at the region where the VRC will remain, and not due to the transport of preexisting PC in the cellular interior^25^. Thus, the 3 PCs identified herein as characteristic molecules for the group of infected patients (Table 1) are putatively related to the pool of PCs synthesized by the infected cells for viral replication. These data are not only in agreement with the role of PCs during viral infection by positive-chain RNA viruses^6,11,25^, but also demonstrate that the results obtained by direct *in vitro* analysis are corroborated *in vivo*.

Since DENV controls the lipid metabolism of the host cell^20,24^, and there is a urge for an additional bioenergetic demand in the viral replication process, the virus promotes the mobilization and recruitment of lipid droplets responsible for the cellular stock of TGs and cholesteryl esters^27^. Recruited TGs undergo the action of lipases in order to provide the necessary fatty acids (FA) for the additional energy supply, since these TGs are used for the production of ATP through the β-oxidation pathway^28^. Cholesterol, also released during this process, will be used to form VRC for viral replication, as well as PCs^29–31^. Thus, TGs identified in this study (Table 1) were indicated as markers present in serum of patients infected with DENV, since the increased bioenergetic demand for viral replication leads to a higher recruitment of TGs as an energy source^24^.

## The role of platelet activation factor in DENV infection

**Figure 3:**
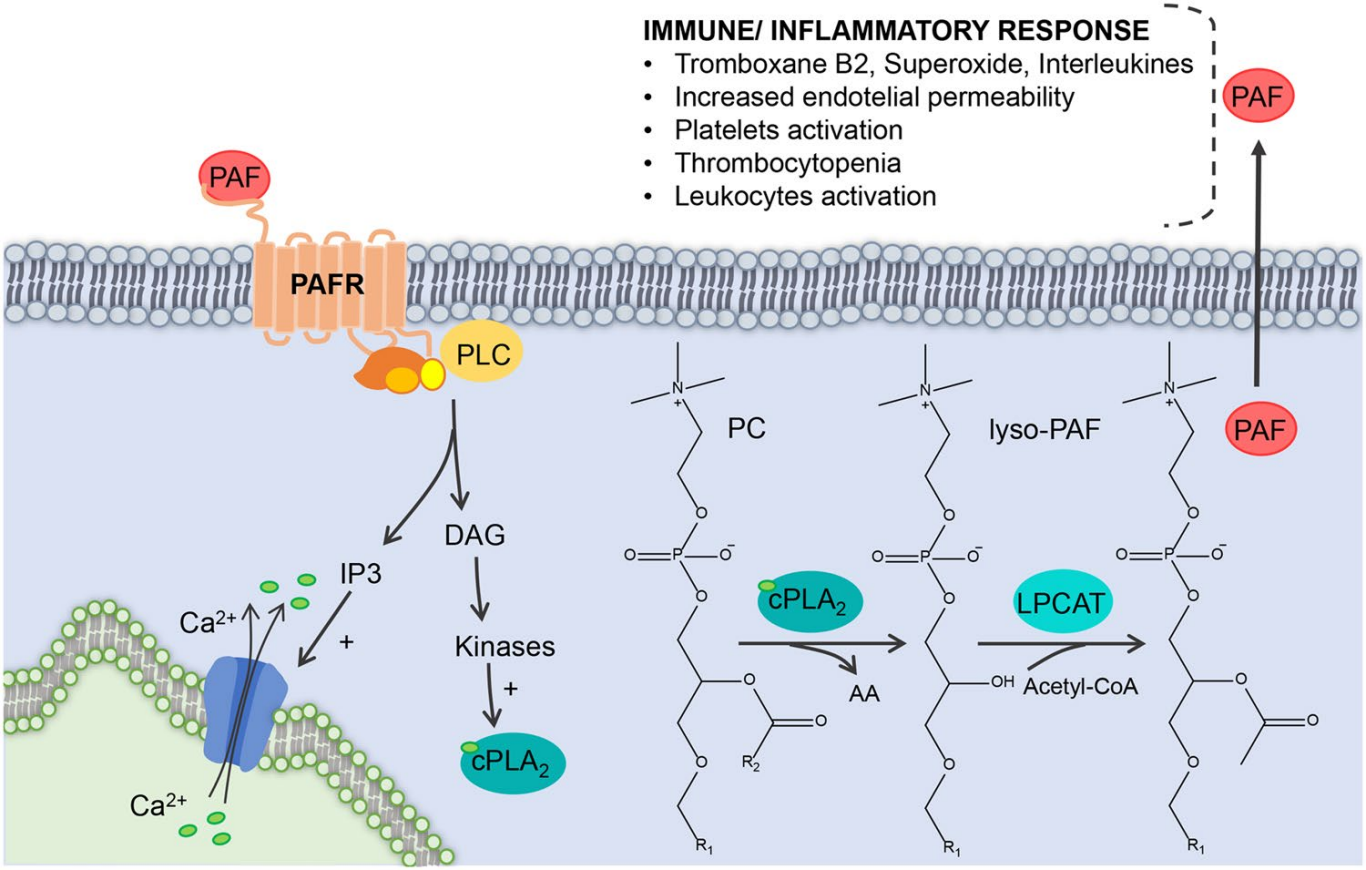
The pool in the synthesis of PAFs that occurs under infection it is mediated by the activation of cPLA2, which recruits and hydrolyzes phosphatidylcholines for the formation of lysoPAF. The activation of cPLA2 due to extracellular stimuli (intracellular phosphorylation and Ca2+ influx) upon PAFs synthesis is initiated by the formation of lyso-PAF. Also, the action of the PAF acetylhydrolase, activated by Ca2+, and phosphorylation of lyso-PAF leads to PAF formation. PAF, Platelet activation factor; PAFR, Platelet activation factor receptor; PLC, Phospholipase; DAG, Diacylglycerol; IP3, Inositol trisphosphate; Ca2+, Calcium ions; cPLA2, Cytosolic phospholipase A2; PC, Phosphatidylcholine; AA, Arachidonic acid; Lysp-PAF, 1-O-alkyl-sn-glycer-3-phosphocholine; LPCAT, Lysophosphatidylcholine acyltransferase; Acetyl-CoA, Acetyl coenzyme A.

Platelet activation factor (PAF) is the trivial name of a phospholipid that has the chemical structure of 1-O-alkyl-2-acetyl-sn-glycero-3-phosphocholine, characterized by an alkyl ether bond at the sn-1 position in the glycerol chain (Fig. 3)^32^. The hexadecyl (16:0) moiety as the linker at the sn-1 position provides greater biological activity to the PAF; however, chain length specificity is low, and this leads to the natural formation of a significant amount of 1-O-octadecyl species, i.e. with an octadecyl (18:0) moiety as the linker at the sn-1 position^33^. Given the variation in the length of the side chain of O-alkyl bound at the sn-1 position, as well as the variation of the 2-acetyl chain attached at the sn-2 position, a series of different PAFs may be formed at the same time^34,35^. The formation of PAFs occurs either by *de novo* synthesis, or by the lipid remodeling pathway^36^. The formation of PAFs by *de novo* synthesis is related to the maintenance of the physiological concentrations of this mediator when the organism is in homeostasis, i.e. constitutive PAF concentrations^37^. The synthesis of PAFs from the remodeling pathway, on the other hand, is regulated by extracellular stimuli, i.e. under inflammation or infection, and is responsible for the PAF pool that occurs under these conditions^38,39^. The synthesis of a pool of PAFs occurring under infection/inflammation is mediated by the activation of cytosolic PLA2 (cPLA2), which recruits and hydrolyzes phosphatidylcholines for the formation of lysoPAF^38,39^. cPLA2 is a member of a superfamily of phospholipases responsible for the recruitment of a series of lipids involved in inflammatory and immune response processes^10^. Therefore, with the activation of cPLA2 due to extracellular stimuli (phosphorylation and Ca^2 +^), PAFs synthesis is initiated by the remodeling pathway, with the formation of 1-O-alkyl-sn-glycer-3-phosphocholine (lyso-PAF) from the hydrolysis of 2-Acyl-1-alkyl-sn-glycero-3-phosphocholine by cPLA2, which has the characteristic of hydrolyzing fatty acids linked to the sn-2 position of the glycerol chain^37^. Thus, the action of the platelet-activating factor acetylhydrolase, activated by Ca^2+^, and phosphorylation of lyso-PAF^40^, leads to PAF formation (Fig. 3).

PAFs exhibit biological activity in various cells and tissues^41^ and the interaction of PAF agonists occur through the PAF receptor (PAFR). PAFR is comprised of seven transmembrane helixes coupled to the G protein, and is present in both plasma membrane and nuclear membrane. Activation of this receptor leads to the influx of Ca^2+^ into the cell, promoting a series of simultaneous signaling of kinases and phospholipases, such as MAPK, PKC, phosphatidylinositol-3-kinase (PI3K), protein tyrosine kinase (PTK), phospholipase Cβ (PLC β), and PLA^42^. Thus, cPLA2 itself is activated by increasing Ca^2+^ concentrations and phosphorylations promoted by the interaction of PAF with its receptor, generating a positive feedback for the production of a PAF pool^40,43^.

The simultaneous activation cascade resulting from PAF promotes a multitude of effects involved in the immune and inflammatory responses, such as the production of superoxide, thromboxane B2 and leukotriene C4^44^, increased endothelial permeability^45^, increased production of proinflammatory interleukins^46^, eosinophils recruitment^47^, among other effects involved in the immune response against viral infection. PAFR is present in the membranes of various cell types such as eosinophils, leukocytes, macrophages, neutrophils and platelets^43^. The correlation between PAF and platelet activation is important in the viral infection process, especially for DENV; the endogenous release of PAF is related to a number of acute inflammation effects in DENV infection, such as increased vascular permeability, altered leukocyte numbers, thrombocytopenia, and degrees of bleeding^48^. Thus, the identification of 4 PAF precursors as biomarkers becomes a strong indication for representing this process.

It has recently been shown that platelets are also involved in the detection of pathogen-associated molecular patterns (PAMPs)^49^ by standard-recognition receptors (PRRs) on the surface, such as Toll-like receptors (TLRs), and associated with other members of the interleukin-1 (IL-1)-TLR superfamily. Moreover, it has been reported that the number of constitutive PRR on the surface of the platelet undergoes specific upregulation when the platelet is active by a given microorganism^50^, which would increase the sensitivity to the immune response to the pathogen, assisting in the fight against infection. This mechanism indicates that platelets are part of the first-line recognition process for both microbial pathogens and immune response. Given the fact that platelets have direct antimicrobial functions mediated by the secretion of antimicrobial molecules, including platelet microbicidal proteins (PMPs) and kinocidins^49^; Tsegaye *et al*. 2013 demonstrated that the release of CXCL4 by platelets inhibited *in vitro* HIV-1 infection in T cells. Thus, it is feasible to infer that platelet activation may play an important role during the viral immune response process^51^.

The identification of precursor species involved in the synthesis of PAFs in this study is in line with the findings proposed by Berthet *et al*. 2012, where peripheral blood platelets exposed to *S. minnesota* led to increased concentrations of IL-6, IL-8 and TNFα, whereas platelets exposed to *E. coli* did not^52^. This behavior evidences that the secretion of platelet cytokines is distinct due to the activation pattern, and suggests a specific response where lipids are responsible for such specificity^53,54^.

In contrast to helping fight viral infections, one of the major complications manifested as a result of the interaction between viral infection and platelets, and which is directly related to the activation of PAF, is thrombocytopenia^55,56^. This characteristic manifestation of DENV infection is even used as one of the criteria for the diagnosis of this infection^56^. Thrombocytopenia has been used as a parameter for the identification of patients with a more severe clinical picture, which also encompass other symptoms such as increased vascular permeability and hematocrit, alterations in the number of leukocytes and hemorrhage (varying degrees)^48^. A study by Yang *et al*. demonstrated greater release of PAF by macrophages in patients with DENV-1 virus than control subjects^57^. In addition, in studies with mice deficient in PAFR, primary infection by DENV was less severe. This occurs because inhibition of the PAF-PAFR interaction decreases the production of proinflammatory cytokines and TNF-α, in addition to decreasing vascular permeability^57,58^. The increase in intracellular Ca^2+^, which may be promoted by the PAF-PAFR interaction, is a crucial factor in the activation of platelet response, including the translocation of P-selectin to the membrane^59^.

Thrombocytopenia in DENV infection occurs due to the immunological destruction of virus-platelet complexes; viral activation of platelets induces overexpression of P-selectin, functioning as a receptor for macrophages^60^. In cases of recurrent DENV infections, antibodies against the prM structural viral protein facilitate efficient binding of their immature particles to cells expressing the Fc receptor, such as platelets (FccRIIa), which bind to these anti-prM-DENV complexes susceptible to destruction by the immune system^61^. In addition, the DENV-platelet complex also binds to complement C3 molecules and to platelet-associated IgM or IgG antibodies, resulting in their clearance by immune system cells^62^, which would result in thrombocytopenia. Additionally to the mechanisms of destruction of the platelet-DENV complex mentioned above, anti-DENV antibodies react against platelet glycoproteins mediating their destruction by the complement or monocyte-macrophage system^63^.

Therefore, all PAF precursors identified and elucidated in this study highlight the importance of increasing the synthesis of PAF performed by the remodeling pathway in the inflammatory process, confirming its activation by external factors^36^. In addition, the synthesis of PAF pools represents the extensive activation of PAFR, a factor related to the severity of dengue cases, which leads to increased cytokines, increased vascular permeability and, consequently, severe hemorrhage and shock^48^. Moreover, platelet activation correlates with the initiation and maintenance of the immune response, as platelets participate in the front line detection and initiation of the immune response^49^. Platelets still play an ambiguous role in the literature, in which they collaborate in the fight against the infection in the organism^49^, while at the same time are involved in the degree of severity of the disease, being responsible for provoking hemorrhagic fever due to thrombocytopenia^56^.

A previous contribution by our research group has confirmed that it is possible to carry out a study on viral infection mechanism through the direct analysis of the serum of infected patients^7^, assertively providing the metabolomics profile of the pathophysiology of the viral infection process, without further degrees of sample preparation and (pre-)processing. Remarkably, this is possible thanks to the integration between mass spectrometry and bioinformatics to analyze the large amount of data generated. All biomarkers were chosen and validated by statistical analysis and are in line with previous studies on the changes arising from DENV infection both *in vivo* and *in vitro*^19,20,25,64^. Thus, the use of these biomarkers opened the possibility to systemically assess the alterations on the lipid pool due to DENV infection, which occurred through the increase of PC synthesis and the recruitment of TGs to supply the bioenergetic needs due to the infection. This study also clarifies the possible mediators of the most severe form of the disease, the hemorrhagic form, since the direct analysis of serum allowed to identify a series of precursors of PAFs. According to data in the literature, the increase in PAFs is closely related to two of the main characteristics of this infection: hemorrhagic fever^16^ and thrombocytopenia^59^. These symptoms, nonetheless, are not exclusively a result of DENV infection: they occur in other infectious diseases as well^55,56,61^. Hence, future efforts in identifying metabolites directly related to several infection processes, as performed herein, will allow us to verify whether the mechanism involved in these diseases is common or specific for each pathogen. Also, our results may enables and encourages the medical community to screen patients with conditions that have potential for hemorrhagic aggravations with a higher degree of confidence for clinical prognosis.

## Methods

### Patients

In this study, serum samples stored in the Research Laboratory of Virology from the Faculty of Medicine of São José do Rio Preto (SJRP), a city located in the northwestern region of São Paulo State, Brazil, were analyzed. All 20 sera samples infected with DENV were obtained from febrile patients serviced in healthcare centers in SJRP during the year of 2014, when Zika virus and Chikungunya virus were not detected in São Paulo State. The control group was composed of 10 healthy adults, i.e. asymptomatic individuals who did not present any signs of infection within 30 days prior to sample collection and presented a negative result in RT-PCR for DENV. All sera were transported in dry ice to the INNOVARE Biomarkers Laboratory in Campinas, SP. This study was conducted according to the Declaration of Helsinki and was approved by the Ethics Committee from the Faculty of Medicine of SJRP (FAMERP), São José do Rio Preto, São Paulo, Brazil (Process Number n° 02078812.8.0000.5415). The collected specimens from all participants consisted of blood samples. Table 2 organizes the structure of sample collection and provides a view of the total number of analyzed specimens. A written informed consent was obtained from all patients prior to enrollment. All samples were obtained from the Base Hospital of SJRP. All experiments were performed in accordance with relevant guidelines and regulations regarding samples from human origin.

**Table 2:** Demographics and clinical conditions of all recruited and included individuals in the study.

Parameters	Groups	
	Control	DENV
RT-PCR exam	Negative	Positive
Symptomatic	No	Yes
**Demographics**		
Male	6	8
Female	4	12
Mean age (median)	32.76 (30)	41.4 (42)
**Days between onset of DENV symptoms disease and sample collection**		
Day 0	NA^a^	6
Day 1	NA^a^	5
Day 2	NA^a^	5
Day 3	NA^a^	4

^a^NA: *Not applicable*.

### DENV detection

All clinical samples used in this study were positive for DENV-NS1 antigen using the NS1 Ag rapid assay kit according to the manufacturer’s instructions. In addition, all samples were positive for DENV by a specific RT Multiplex-Nested-PCR performed after RNA extraction from 140 uL of serum with the QIAamp Viral RNA mini kit (QIAGEN), according to the manufacturer’s protocol. The Multiplex-Nested-PCR to DENV 1–4 were performed according Colombo and collaborators, 2016^65^.

### Sample preparation

Serum preparation was performed as described by Melo *et al*. 2017^7^. In summary, 20 μL of each biological sample (blood serum) were diluted in 200 μL of tetrahydrofuran and homogenized and then the volume was completed to 1 mL with methanol, with further homogenization. The obtained solution was centrifuged and 20 μL of the supernatant was collected and diluted in 980 μL of methanol and 0.1% of formic acid was added to the final solution.

### High Resolution Mass Spectrometry Analysis

All samples were directly injected for survey scan analysis in an ESI-LTQ-XL Orbitrap Discovery instrument (Thermo Scientific, San Jose, California) with nominal resolution of 30,000 (FWHM), under the following parameters: flow rate of 10 μL.min^−1^, capillary temperature of 280 °C, 5 kV as spray voltage and sheath gas at 10 arbitrary units. HRMS analyses were performed in technical quintuplicates for each sample using the mass range of 500–2000 *m/z* in the positive ion mode. Spectra were analyzed using XCalibur software (v. 2.4, Thermo Scientific, San Jose, CA)

### Statistical analysis

Statistical analysis to choose chemical markers for each group was performed using Principal Component Analysis (PCA). PCA is a multivariate model of covariance structure modeling; it is used with the specific purpose of analyzing correlation structures, and it is characterized as a statistical analysis technique for potential biomarkers screening by a given “omic” platform^14^. To perform PCA analyses, raw data were used as a pool of all samples within the same data matrix, i.e. all mass spectrometric data from all replicates of both Control and DENV group were organized in a single database, which was inputted in the online platform environment. Prior to PCA analyses, interquartile range was used as data filtering method, with quantile normalization and auto scaling. A heatmap of the all features selected by PCA analyses was built using the Pearson’s distance measurement and Ward’s clustering algorithm. Fold Change analysis was performed for all features selected by PCA and elucidated by HRMS and MS/MS analysis. All statistical analyses were performed using the online platform MetaboAnalyst 3.0^66,67^.

### Identification of markers

METLIN (Scripps Center for Metabolomics, La Jolla, CA) was consulted to elect the most suitable markers based on the exact mass of each species, adopting a maximum error of 2 ppm for mass accuracy from the experimental exact mass obtained in the study and adducts of [M + H^+^] and [M + Na^+^] available on the platform^15^. The markers selected on METLIN were confirmed across MS/MS data acquired in the same instrument used for the HRMS analyses and with the same setup. MS/MS reactions were carried out using Helium as the collision gas, with energies for collision-induced dissociation (CID) ranging from 16 to 31 (arbitrary units). The fragmentation analysis profile spectra of MS/MS were analyzed using XCalibur software (v. 2.4, Thermo Scientific, San Jose, CA) and structures were confirmed using theoretical calculations modeling for molecular fragmentation using Mass Frontier software (v. 6.0, Thermo Scientific, San Jose, CA) (Table 1 and Supplementary Information 1).

## Electronic supplementary material

Supplementary Information
