# Supplementary material for: The role of lipids in the inception, maintenance and complications of dengue virus infection
Source: Sci Rep. 2018 Aug 7;8:11826. doi: 10.1038/s41598-018-30385-x (PMC6081433; doi:10.1038/s41598-018-30385-x)

The role of lipids in the inception, maintenance and complications of dengue virus infection

Carlos Fernando Odir Rodrigues Melo[1], Jeany Delafiori[1], Mohamad Ziad Dabaja[1], Diogo Noin de Oliveira[1], Tatiane Melina Guerreiro[1], Tatiana Elias Colombo[2], Maurício Lacerda Nogueira[2], Jose Luiz Proenca-Modena[3], Rodrigo Ramos Catharino[1]*

[1]INNOVARE Biomarkers Laboratory, School of Pharmaceutical Sciences, University of Campinas, Brazil.
[2]School of Medicine from São José do Rio Preto (FAMERP), Brazil.
[3]Laboratory of Study of Emerging Viruses (LEVE), Department of Genetic, Evolution and Bioagents, Institute of Biology, University of Campinas, Brazil.

* Corresponding author: Rodrigo Ramos Catharino

Supplementary Information #1

MS/MS Reactions of the Species Elected by PCA

MS/MS spectrum of 743 *m/z*

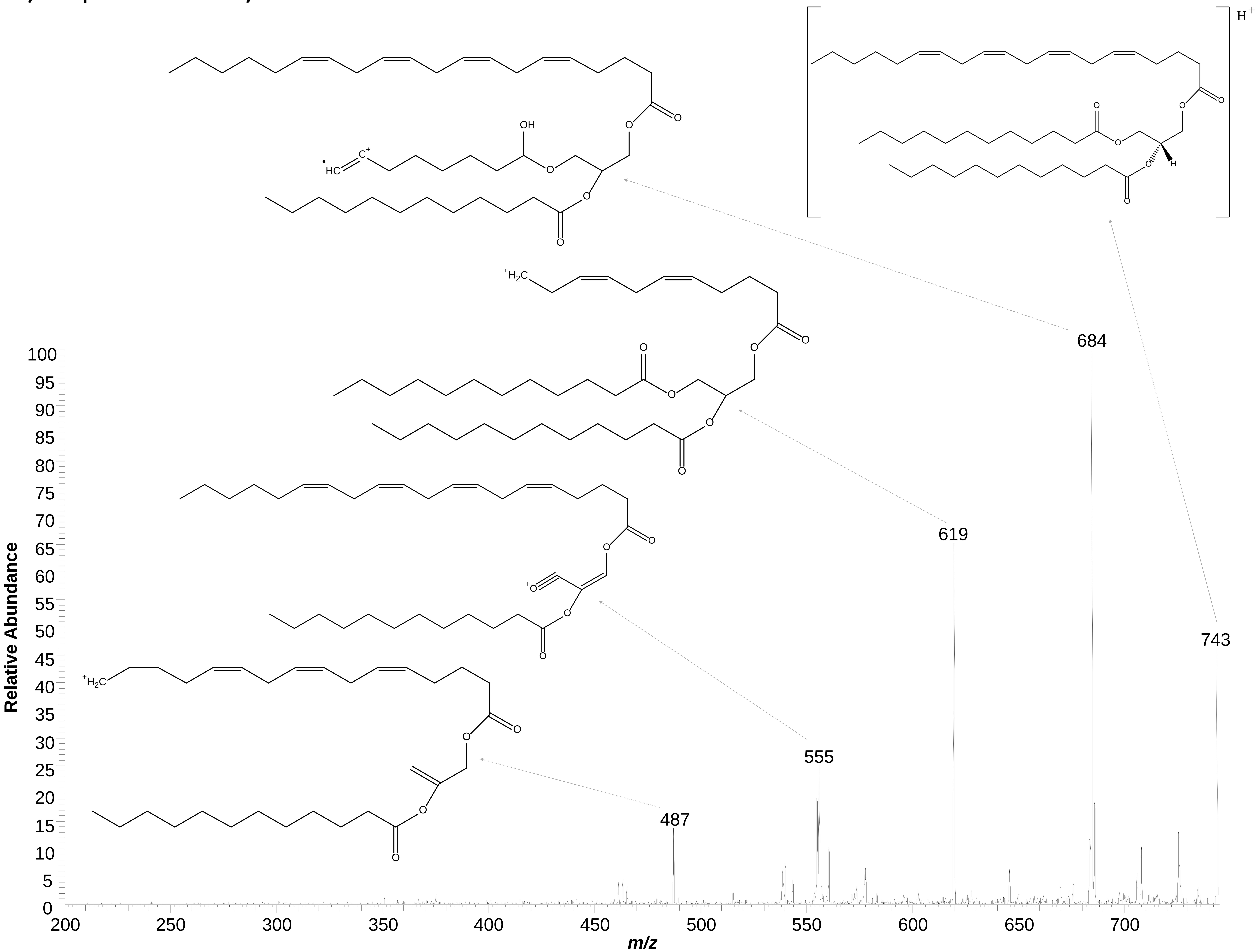

MS/MS spectrum of 769 *m/z*

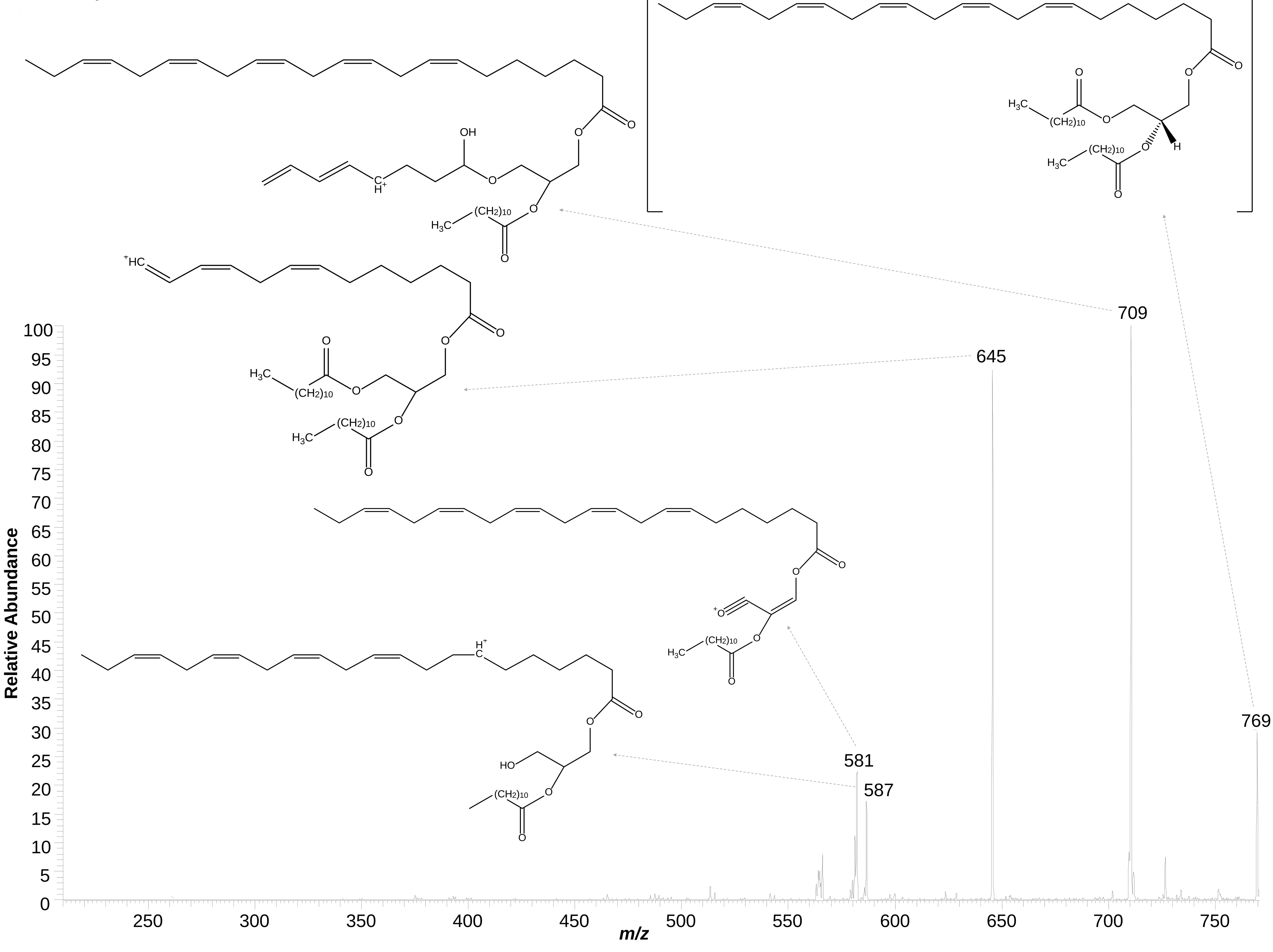

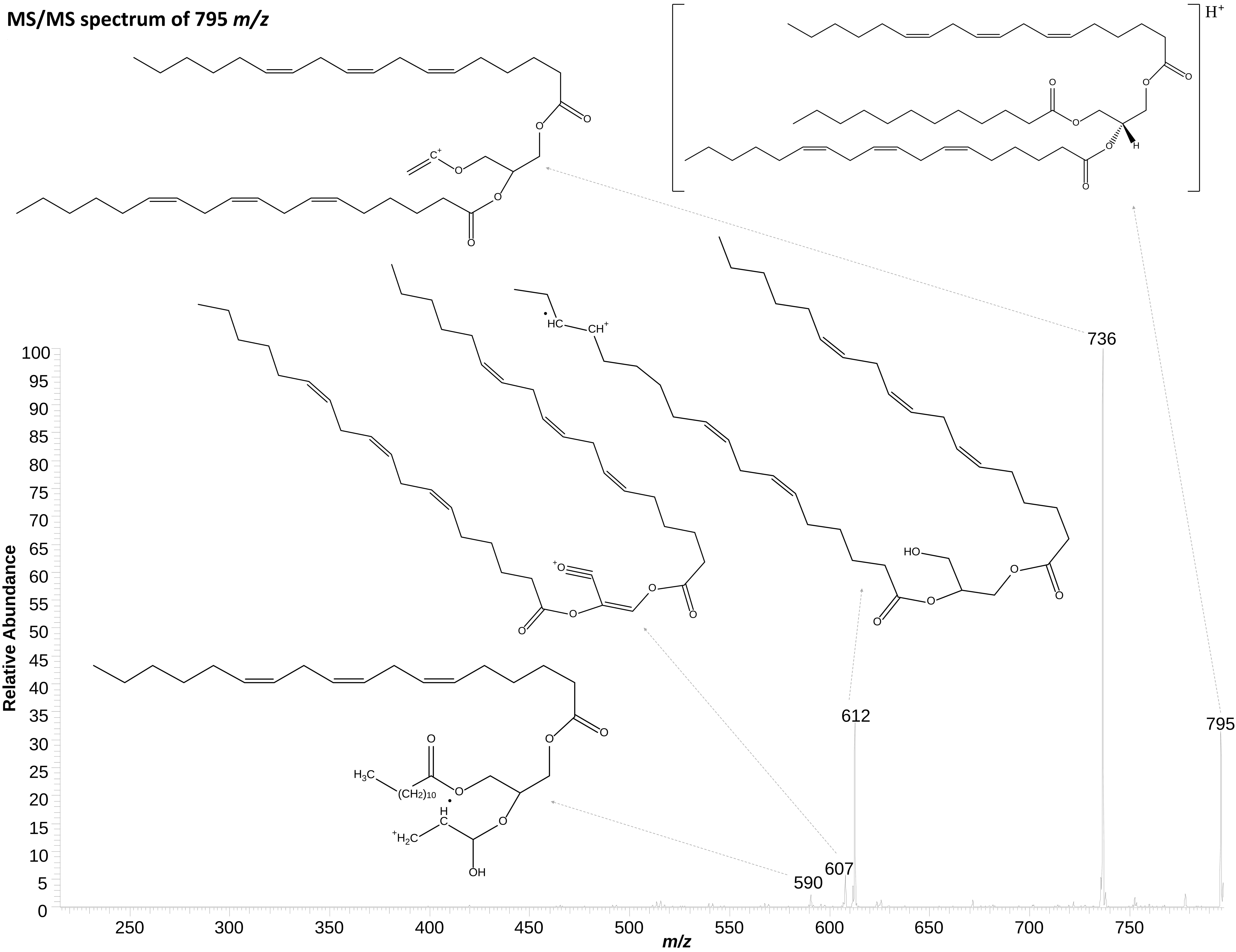
MS/MS spectrum of 795 m/z
H+
736
612
795
590
607
Relative Abundance
m/z
HO
OH
H3C
(CH2)10

MS/MS spectrum of 859 *m/z*

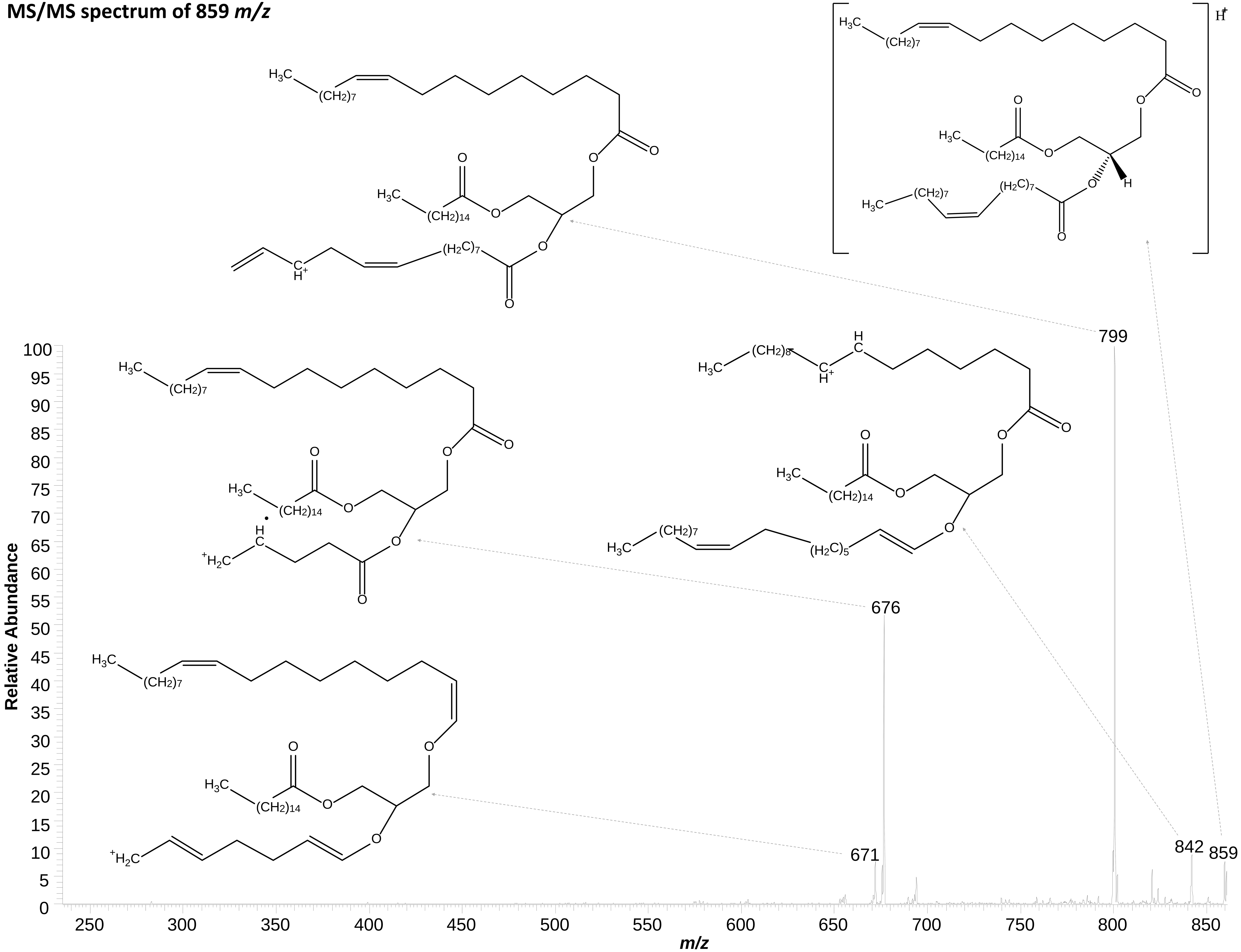

MS/MS spectrum of 762 *m/z*

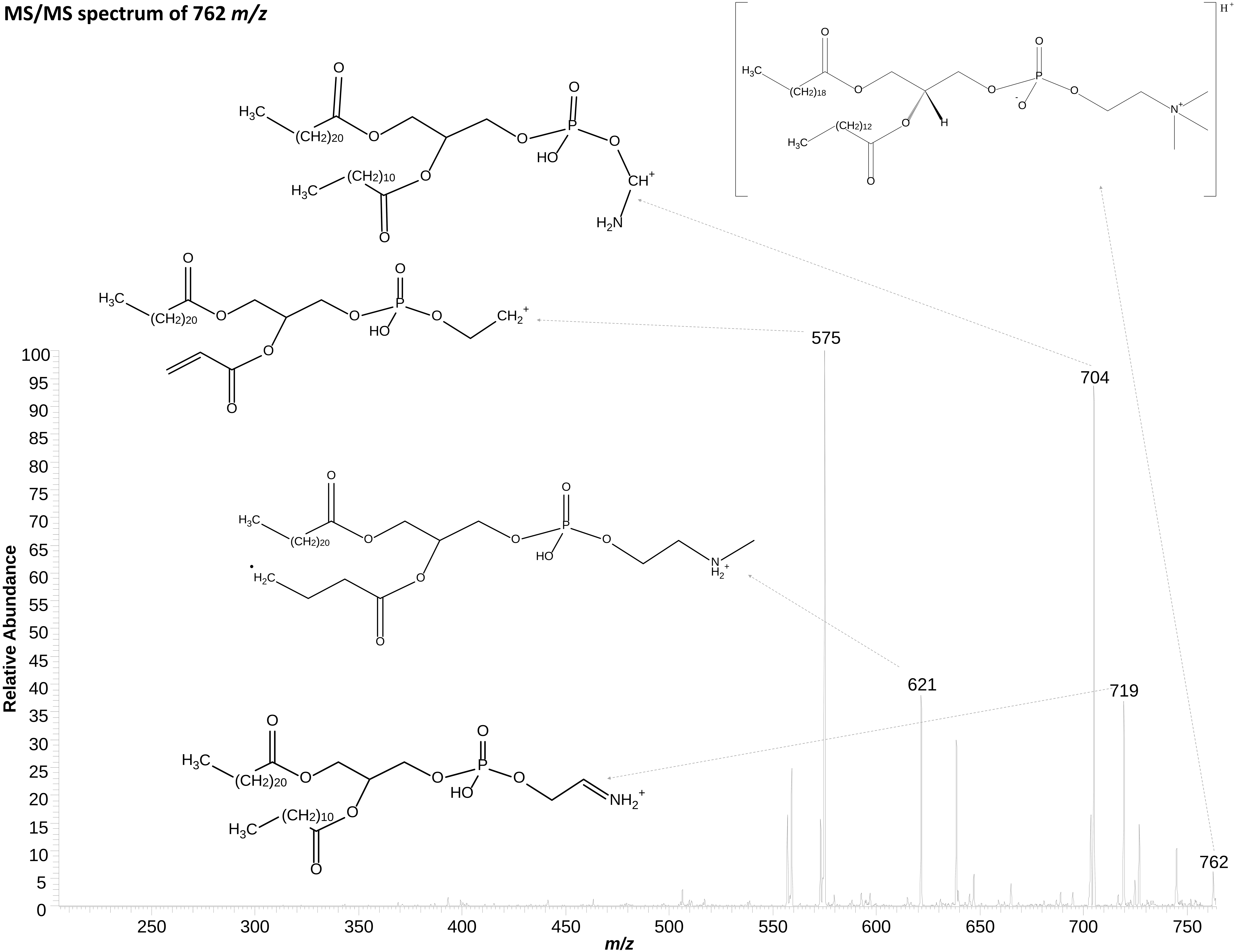

MS/MS spectrum of 784 *m/z*

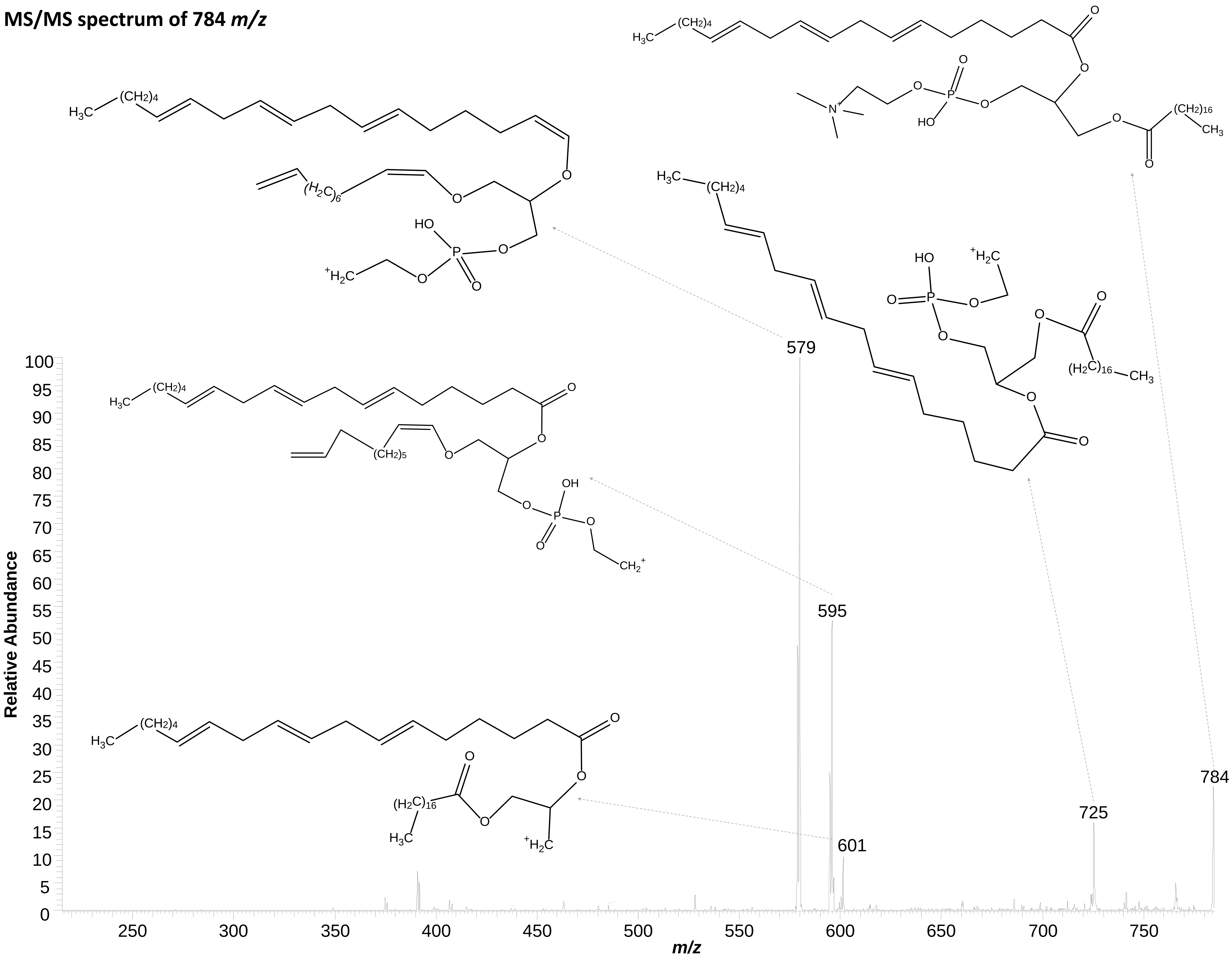

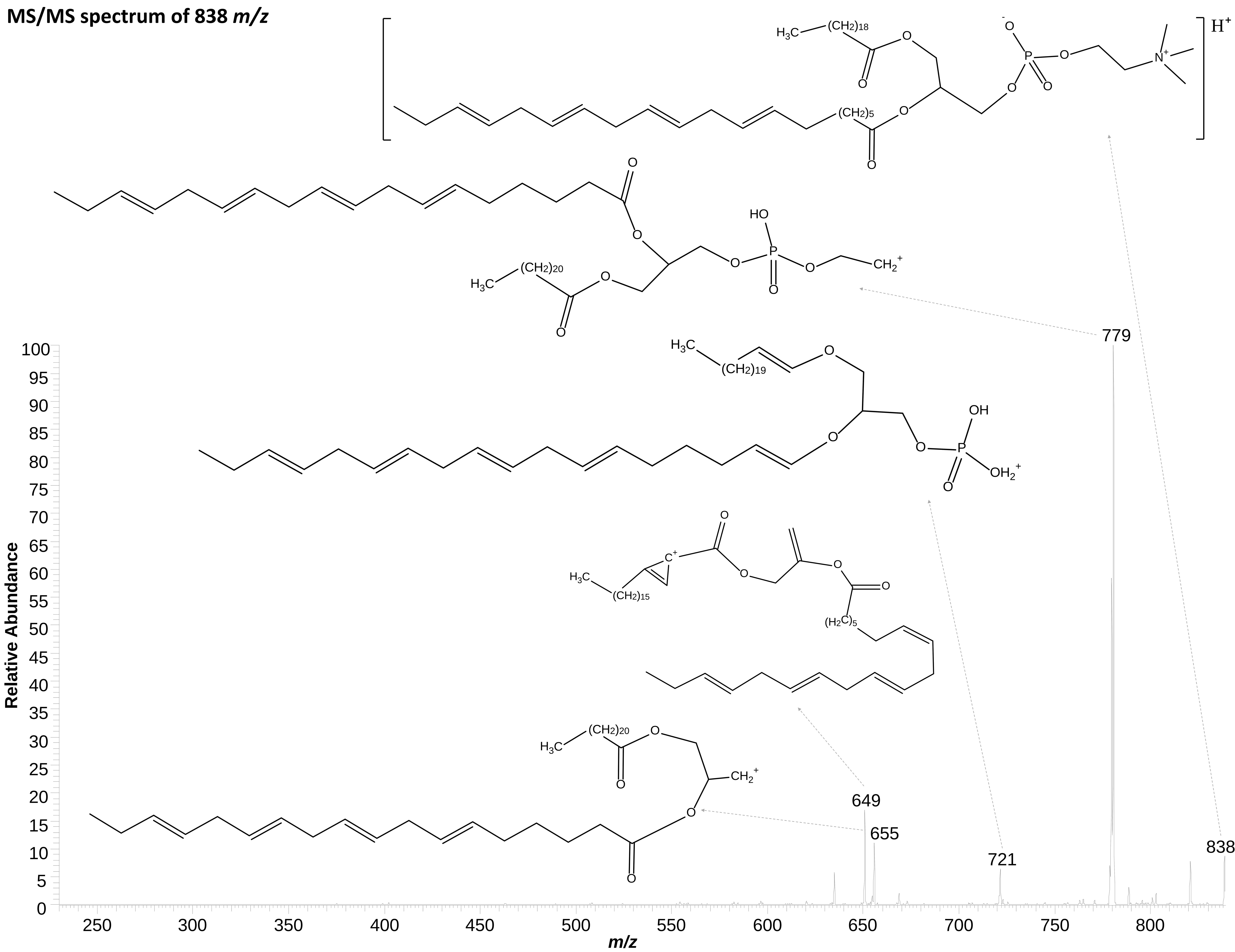

MS/MS spectrum of 838 m/z
H+
779
649
655
721
838
Relative Abundance
m/z
0
5
10
15
20
25
30
35
40
45
50
55
60
65
70
75
80
85
90
95
100
250
300
350
400
450
500
550
600
650
700
750
800

MS/MS spectrum of 768 *m/z*

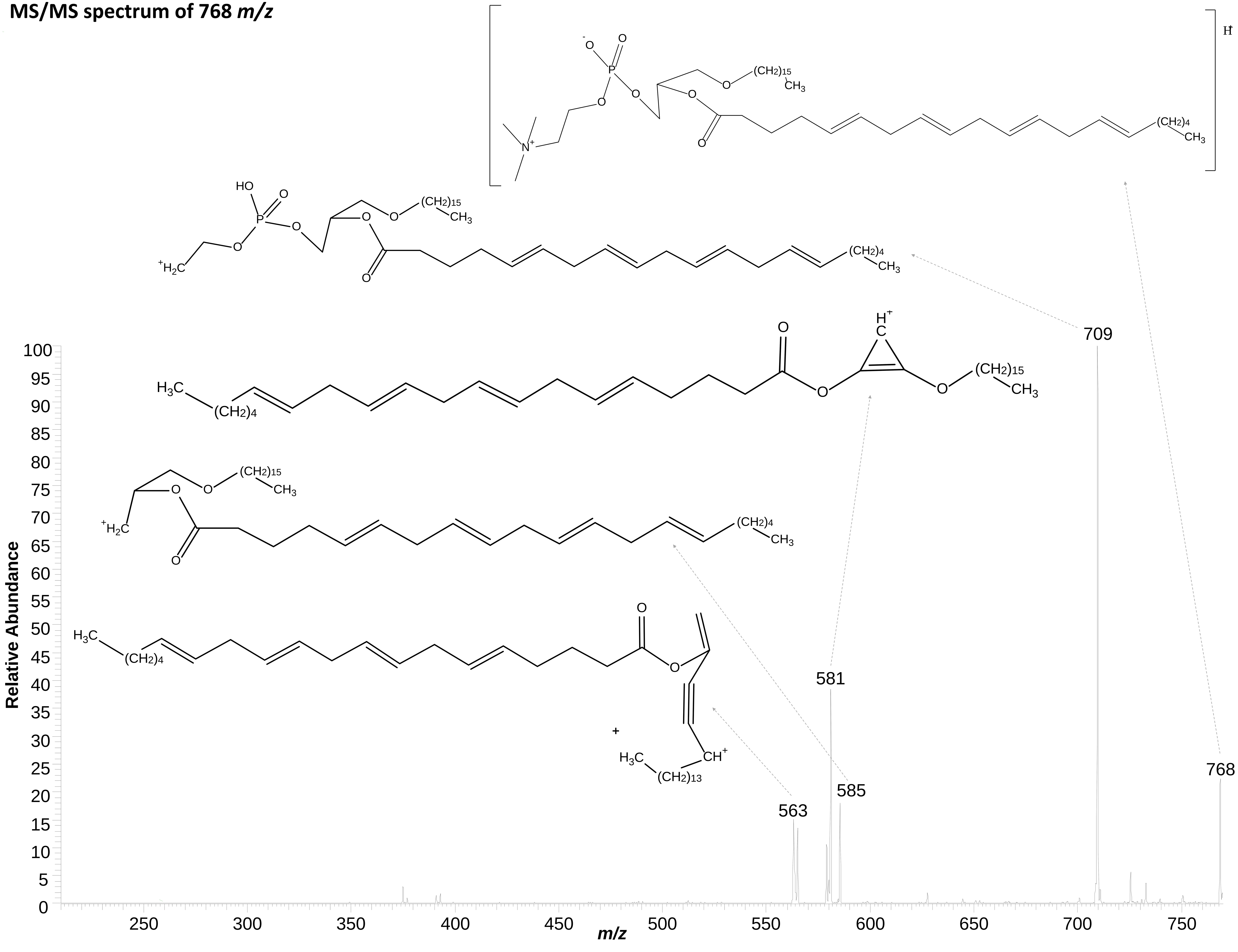

MS/MS spectrum of 770 *m/z*

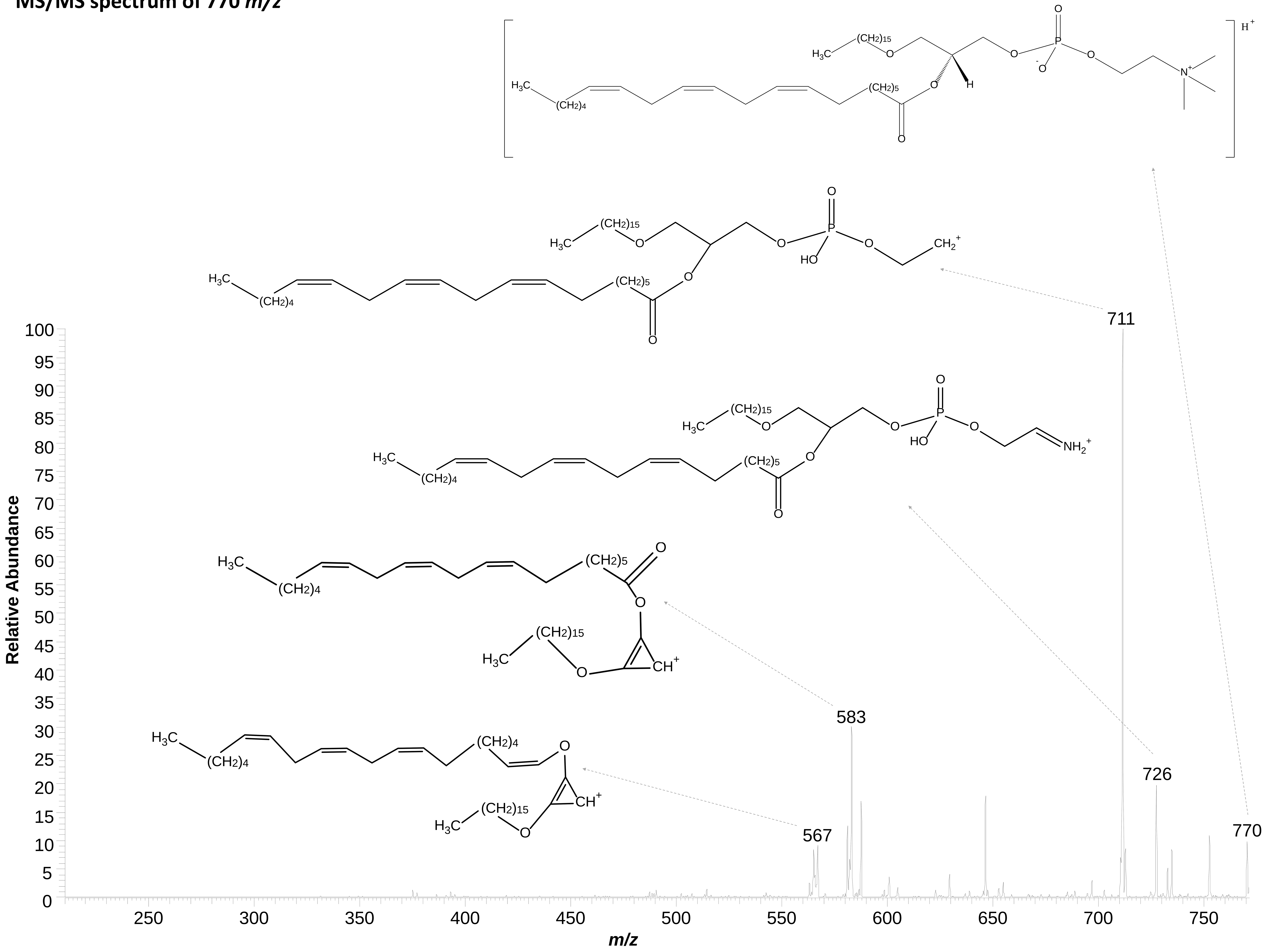

MS/MS spectrum of 792 *m/z*

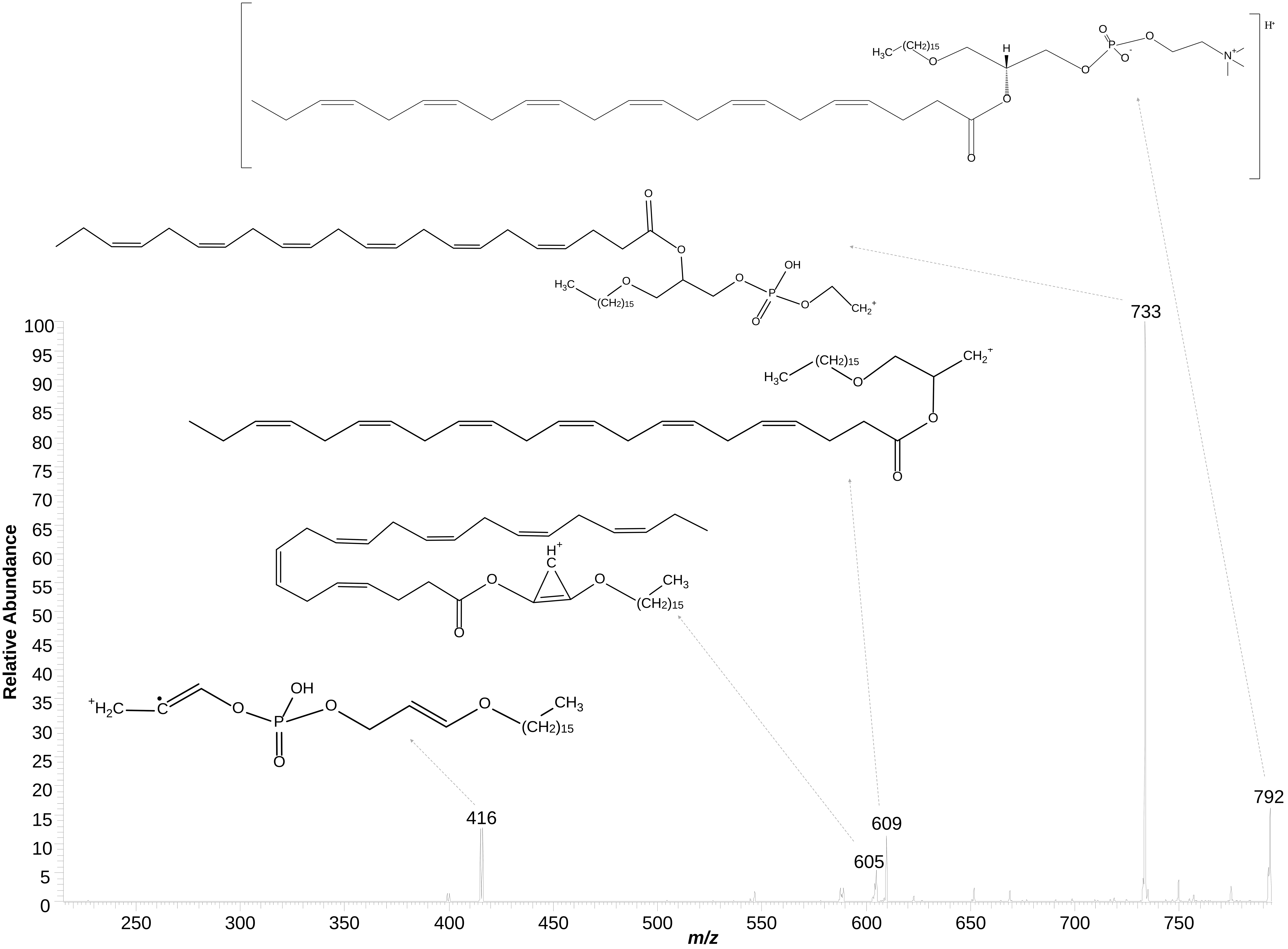

MS/MS spectrum of 798 *m/z*

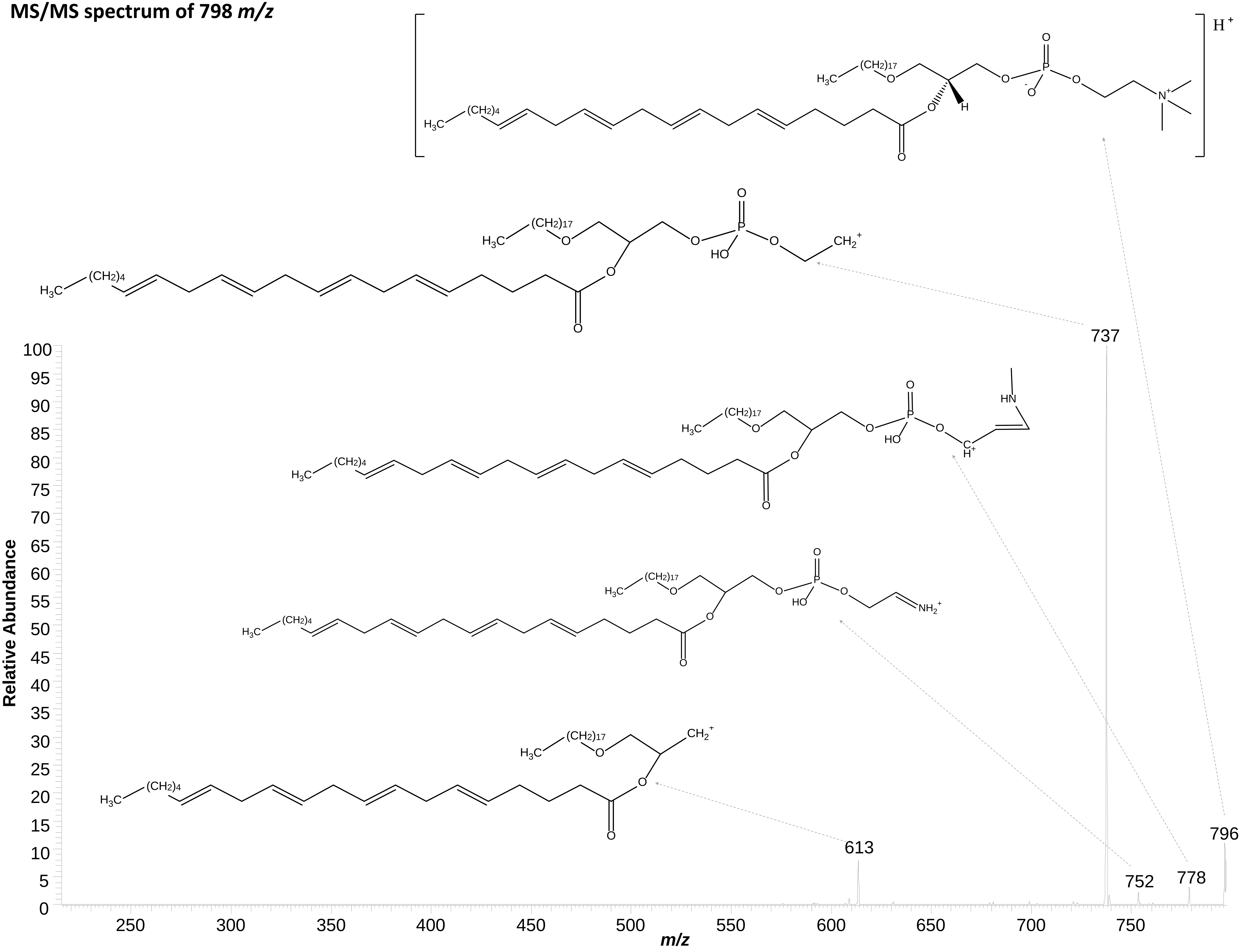

Supplement: Supplementary file 1 — Supplementary Information [file 41598_2018_30385_MOESM1_ESM.pdf]
